# Central Sleep Apnoea and Arrhythmogenesis After Myocardial Infarction—The CESAAR Study

**DOI:** 10.3389/fcvm.2019.00108

**Published:** 2019-08-06

**Authors:** Alexander Reshetnik, Swetlana Puppe, Hendrik Bonnemeier

**Affiliations:** ^1^Charité - Universitätsmedizin Berlin, Corporate Member of Freie Universität Berlin, Humboldt-Universität zu Berlin, and Berlin Institute of Health; Department of Nephrology, Berlin, Germany; ^2^Department of Psychiatry, Evangelisches Krankenhaus Königin Elisabeth Herzberge gGmbH, Berlin, Germany; ^3^Department of Cardiology, Universitätsklinik Schleswig-Holstein, Kiel, Germany

**Keywords:** central sleep apnoea, sleep disorder breathing, acute myocardial infarction, non-sustained ventricular tachycardia, arrhythmogenesis

## Abstract

The prevalence of sleep disordered breathing (SDB) after acute myocardial infarction (AMI) is high. However, little is known about predominant SDB type and the impact of SDB severity on arrhythmogenesis. We conducted a prospective single-center observational study and performed an unattended sleep study and Holter monitoring within 10 days after AMI, and an unattended sleep study 11.3 months after AMI. All patients were included from the Department of Cardiology at the University Hospital Schleswig-Holstein, Lübeck, Germany. A total of 202 subjects with AMI (73.8% with ST-elevation; 59.8 years; 73.8% male) were included. The mean BMI was 27.8 kg/m^2^ and the mean neck/waist circumference was 41.7/103.3 cm. The mean left ventricular ejection fraction was 56.6%. The SDB prevalence defined as apnoea-hypopnea-index (AHI) ≥ 5/h was 66.7% with 44.9% having central (CSA), and 21.8% obstructive sleep apnoea (OSA). The mean AHI was 13.8 1/h. In 10.2% nsVT was detected in the Holter monitoring. AI >23/h was independently associated with higher risk of nsVT in the subacute AMI period. SDB is highly prevalent and CSA a predominant type of SDB in the subacute phase after uncomplicated AMI treated with modern revascularization procedures and evidence-based pharmacological therapy. Severe SDB is independently associated with higher risk for nsVT in the subacute AMI period and its course should be monitored as it can potentially have a negative impact on relevant outcomes of AMI patients. Further prospective studies are needed to assess long-term follow up of SDB after AMI and its impact on mortality and morbidity.

## Introduction

Ischemic heart disease is one of the major causes of death worldwide. Introduction and widespread use of modern revascularization procedures and evidence-based pharmacological therapy improved the long-term prognosis after acute myocardial infarction (AMI) significantly (1, 2). However, strategies to further improve the outcome of patients with AMI are highly welcome. One strategy is to focus on the impact of comorbidities, such as sleep-disordered breathing (SDB). SDB results in intermittent hypoxia (3), stress and sympathetic activation, which can negatively influence the outcome of patients after AMI through induction of myocardial oxidative stress (4), additional ischemic injury, and limited recovery of left ventricular ejection fraction (LVEF) (5). Several epidemiological studies demonstrated high prevalence of SDB after AMI (6–9). Central sleep apnoea (CSA) is a type of SDB common in patients with congestive heart failure (10–12). Several pathophysiological changes such as pulmonary congestion, impaired autonomic function, and sympathetic hyperactivity, which occur after AMI, are known causes of CSA (13, 14). However, reports regarding CSA prevalence after AMI and its impact are scarce (6, 15, 16), which is in part due to simple polygraphy (PG) devices used without the opportunity to differentiate between central, obstructive, or mixed apnoea events. We conducted a prospective single-center study to observe the prevalence of SDB and CSA in particular in the subacute phase of AMI and to assess the impact of SDB severity on the prevalence of non-sustained ventricular tachycardia (nsVT) as a known predictor of sudden cardiac death in post-infarct patients (17, 18).

## Materials and Methods

### Study Collective and Design

This was a prospective observational study. We screened 1,935 patients with AMI, who were admitted to the Department of Cardiology of the University Hospital of Lübeck, Germany, between October 2006, and March 2009. Inclusion criteria were defined as follows: confirmed AMI diagnosis (ST-elevation myocardial infarction and non-ST-elevation myocardial infarction) defined according to the criteria of European Society of Cardiology (19, 20) with a completed coronary angiography, and revascularized culprit vessel. Exclusion criteria were defined as follows: patient not able or not willing to complete written informed consent; hemodynamically or respiratory instable subjects; concurrent therapy with oxygen; current SDB therapy or previously diagnosed SDB based on the findings of a polysomnography; age ≥ 80 years; dementia; severe chronic obstructive lung disease; any current medication able to induce central sleep apnoea; AMI event happened longer than 10 days ago. From the 19,35 eligible patients 202 subjects who fulfilled the inclusion criteria without any of the exclusion criteria were included in the study protocol. All of them completed written informed consent. The ethics committee of the University Hospital of Lübeck, Germany, approved the study.

**Figure 1:**
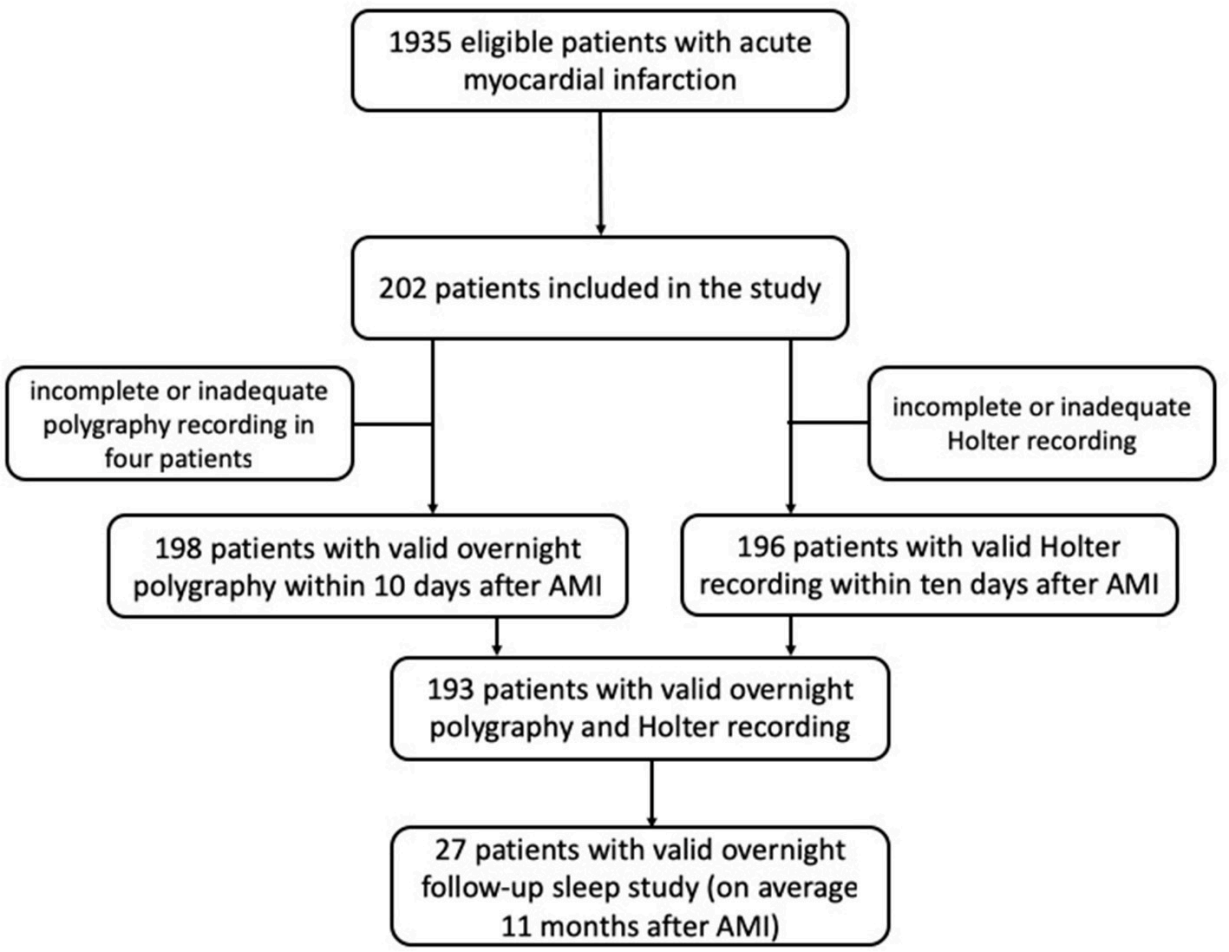
Study flow chat. AMI–acute myocardial infarction.

An unattended overnight sleep cardiorespiratory PG and a Holter electrocardiography (ECG) over 24 h were performed in the subacute phase of myocardial infarction (day 2–10). Follow-up sleep study was performed in the long-term after AMI. Demographic and medical data were collected from medical records and patient interviews. Study schematic is shown in Figure 1.

### Sleep Monitoring

Initial unattended portable sleep monitoring was performed with PolyMESAM® (MAP, Munich, Germany), which is a validated level 3 modified unattended portable sleep monitoring device (21). Heart rate and oxygen saturation were measured by finger pulse oximetry, nasal/oral airflow by thermistor, chest, and abdomen wall motion by inductance plethysmography, and body position were recorded during sleep monitoring. Obtained data were automatically analyzed with the device software in terms of heart rate changes, desaturations, apnoea, and hypopnea events. The findings were validated manually by study investigators (AR, SP), who had received appropriate training in validating the findings of PG, according to the American Academy of Sleep Medicine (AASM) scoring recommendations (22). Event duration was defined as the time from the nadir preceding the first breath being significantly reduced to the beginning of the first breath that approximates the baseline breathing amplitude. A central apnoea event was defined as a complete or almost complete (>90%) cessation of airflow for at least 10 s in the absence of chest and abdomen wall motion. An obstructive apnoea event was defined as a complete or almost complete (>90%) cessation of airflow for at least 10 s with chest and/or abdomen wall effort. A hypopnea was defined as a reduction in airflow of at least 30% from baseline associated with a fall in oxygen saturation ≥ 3% from pre-event baseline for at least 10 s. To express the severity of SDB, we calculated the number of events per hour of sleep and defined as index: apnoea-hypopnea-index (AHI), central (CAI), and obstructive apnoeas per hour [obstructive apnoea index (OAI), and the total number of hypopneas (hypopnea index (HI)]. The total sleep duration was defined as a time with stable breathing pattern in the sleep study. The severity of desaturation events during sleep monitoring was expressed as the number of episodes of desaturation below 90% per hour–oxygen desaturation index (ODI). SDB was diagnosed when AHI was equal to or higher than five events per hour. We considered subjects having CSA when more than 50% of apnoea events were classified as central or having obstructive sleep apnoea (OSA), when more than 50% of apnoea events were classified as obstructive according to AASM criteria. Cheyne-Stokes respiration pattern was defined when at least three cycles of crescendo and decrescendo change in breathing amplitude occurred and CAI was five or more events per hour.

### Holter Monitoring

A 3-lead Holter monitoring (Tracker III, Reynolds, Hertford, UK) was used. Obtained data were automatically analyzed with the device software and validated manually by the experienced study investigator (HB), who was blinded for the results of sleep monitoring. nsVT was considered, when a run of at least three consecutive ventricular beats with a rate more than 100 per min and a whole event duration shorter than 30 s occurred. Sustained VT was considered when the event duration was longer than 30 s.

The prevalence of high-grade tachycardic (nsVT, VT, ventricular fibrillation) and bradycardic (AV block 2° and 3° and asystole) arrythmias was analyzed. Single ventricular extrasystoles were not included in the final analysis. The impact of SDB on the high-grade arrhythmia type with the highest incidence was then analyzed.

### Statistical Analysis

Statistical analysis was conducted with a commercially available software package (SPSS version 13.0; SPSS Inc., Chicago, USA). Continuous variables are shown as mean ± standard deviation (SD). Categorical variables are shown as numbers and percentages. Continuous variables were tested for normal distribution with the Kolmogorov-Smirnov test. *T*-test was used to compare normally distributed continuous variables. In case the normal distribution was not present, Mann-Whitney *U*-test was used for the comparison. Chi square test was used to compare categorical variables. Binary logistic regression was used to assess the impact of SDB on arrhythmogenesis. “nsVT occurrence” was used as a dependent dichotomous variable (“yes” if at least one nsVT was detected in the Holter monitoring and “No,” if no nsVT events were detected in the Holter monitoring). All available demographic, cardiovascular, and sleep study parameters were consecutively inputted in the univariate regression analysis as independent variables. EF was converted in a dichotomous variable as EF <35% (“yes” vs. “no”), as severely impaired EF has major effect on the incidence of CSA. For SDB severity indices with significant impact on nsVT occurrence in univariate tests different cut-off values were further tested for their impact on nsVT occurrence. Parameters which showed significant impact on nsVT occurrence in the univariate tests were entered into multivariate regression analysis, whereby SDB severity indices were inputted as values above the cut-off, which was significant in the univariate test. A two-sided *p*-value of <0.05 was considered statistically significant. No adjustment was made for multiplicity.

## Results

Two hundred and two subjects were included in the study. Mean age of the study collective was 59.8 years and the mean BMI 27.8 kg/m 2.73.8% were male. 56.4% had hypertension, 16.3% diabetes mellitus, 69.3% hyperlipidemia, and 58.4% smoked at the time of study inclusion. Mean measured waist circumference was 103.3 cm and the mean neck circumference was 41.7 cm at the study inclusion. The mean age of SDB-group was 61.7 years and significantly higher than in non-SDB-group (56.7 years). The mean BMI in SDB-group was 28.6 kg/m2 and significantly higher than in non-SDB-group. The prevalence of hypertension was significantly higher in the SDB-group (64.4 vs. 42.4%, respectively). Significantly more subjects smoked in the non-SBD-group (71.2 vs. 50.8%, respectively). The neck and waist circumference were significantly higher in the SDB-group (42.4 vs. 40.4 cm and 105.6 vs. 99.2 cm, respectively). Further baseline characteristics for the whole study collective, SDB- and non-SDB groups, are shown in Table 1.

**Table 1:** Baseline characteristics of the study collective, sleep disordered breathing (SDB) group and non-SDB group.

	**All patients**	**Non-SDB**	**SDB**
	**(*n* = 202)**	**(*n* = 66)**	**(*n* = 132)**
Age, years	59.8 ± 10.8	56.7 ± 9.5	61.7 ± 10.9^¶^
BMI, kg/m^2^	27.8 ± 4.0	26.1 ± 3.3	28.6 ± 4.0^¶^
Men, *n* (%)	149 (73.8)	45 (68.2)	101 (76.5)
History of CAD, *n* (%)	31 (15.4)	5 (7.6)	26 (19.7)
Previous CABG, *n* (%)	7 (3.5)	1 (1.5)	6 (4.5)
Previous MI, *n* (%)	23 (11.4)	4 (6.1)	18 (13.7)
Hypertension, *n* (%)	114 (56.4)	28 (42.4)	85 (64.4)^¶^
Diabetes mellitus, *n* (%)	33 (16.3)	6 (9.1)	27 (20.5)
Smoker, *n* (%)	118 (58.4)	47 (71.2)	67 (50.8)^¶^
Hyperlipidemia, *n* (%)	140 (69.3)	47 (71.2)	91 (68.9)
ß-blocker, *n* (%)	198 (98.0)	65 (98.5)	129 (97.7)
ACE inhibitor, *n* (%)	191 (94.6)	66 (100)	121 (91.7)^*^
ARB, *n* (%)	11 (5.4)	0 (0)	11 (8.3)
Calcium channel blocker, *n* (%)	14 (6.9)	4 (6.1)	10 (7.6)
GBIIbIIIa receptor blocker, *n* (%)	149 (73.8)	48 (72.7)	98 (74.2)
Systolic blood pressure at admission, mmHg	140 [120–150]	140 [120–150]	140 [120–150]
Diastolic blood pressure at admission, mmHg	80 [70–90]	80 [71–85]	80 [70–90]
Neck circumference, cm	41.7 ± 3.7	40.4 ± 3.4	42.4 ± 3.6^#^
Waist circumference, cm	103.3 ± 12.0	99.2 ± 10.6	105.6 ± 12.2^#^
Creatinine, μmol/l	74 [65–87]	72 [64–83]	75 [65–88]
CRP, mg/l	15.2 [5.5–42.2]	11.9 [3.8–30.9]	16.3 [6.9–49.6]
Leukocytes, 1/nl	8.6 [7.4–10.4]	8.6 [7.2–10.3]	8.6 [7.4–10.5]
Troponin T at admission, μg/l	0.20 [0.06–0.54]	0.21 [0.06–0.57]	0.20 [0.06–0.54]
CKmax, U/l	1,255 [494–2,796]	1,369 [294–2,254]	1,243 [543–3,031]
CKMBmax, U/l	133 [45–265]	140 [39-232]	133 [49.5–280]
LDHmax,U/l	384 [224–663]	377 [185–608]	384 [233–690]

Continuous normally distributed variables are presented as mean ± standard deviation; Continuous non-normally distributed variables are presented as median [25% quartile−75% quartile]; ACE, angiotensin-converting enzyme; AHI- apnoea-hypopnea-index; ARB, angiotensin receptor blocker; BMI, body mass index; CABG, coronary artery bypass graft; CAD, coronary artery disease; CK, creatinine kinase; CVD, cardiovascular disease; MI, myocardial infarction;

* p < 0.05;

¶ p < 0.01;

# *p < 0.001; Mann-Whitney U test was used for non-normally distributed data*.

Ninety five percentage of the included patients were treated by primary percutaneous coronary intervention, which included angioplasty and stenting. The majority of included subjects had a ST-elevation myocardial infarction (71.8%). The mean ejection fraction was 55.1%. Subjects in the SDB-group had significantly higher rates of angiographically confirmed Triple-Vessel coronary artery disease (42.4 vs. 23.1%, respectively) and significantly lower rates of Single-Vessel coronary artery disease compared to non-SDB group (22.7 vs. 43.1%). Further specific cardiological characteristics for the whole study collective, SDB-, and non-SDB-group, are given in Table 2.

**Table 2:** Specific cardiological characteristics of the study collective, sleep disordered breathing (SDB) group and non-SDB group.

	**All patients**	**Non-SDB**	**SDB**
	**(*n* = 202)**	**(*n* = 66)**	**(*n* = 132)**
1-vessel-CAD, *n* (%)	59 (29.2)	28 (43.1)	30 (22.7)^¶^
2-vessel-CAD, *n* (%)	69 (34.2)	22 (33.8)	46 (34.8)
3-vessel-CAD, *n* (%)	73 (36.1)	15 (23.1)	56 (42.4)^*^
STEMI, *n* (%)	145 (71.8)	49 (74.2)	92 (69.7)
NSTEMI, *n* (%)	57 (28.2)	17 (25.8)	40 (30.3)
Anterior MI, *n* (%)	88 (43.6)	28 (42.4)	58 (43.9)
Non-Anterior MI, *n* (%)	114 (56.4)	38 (57.6)	74 (56.1)
Culprit lesion vessel, *n* (%)	81 (40.1)	26 (40.6)	54 (40.9)
LAD			
RCA	78 (38.6)	26 (40.6)	51 (38.6)
LCX	41 (20.3)	12 (18.8)	27 (20.5)
Pain-to-ballon time, min	244 [172–600]	223 [150–371]	457[225–520]^*^
Pain-to-ballon time > 24 h, *n* (%)	34 (16.8)	10 (15.5)	22 (16.7)
Angiographic EF, %	56.8 [47.8–64.6]	60.0 [50.3–65.5]	60.0 [50.3–65.5]
Echocardiographic EF, %	55.5 [48.3–63.0]	59.0 [50.0–67.3]	55.0 [48.0–60.0]
Fractional Shortening, %	32.0 [25.0–38.0]	34.0 [27.0–39.0]	30.0 [25.0–36.3]
ESLVD, cm	3.4 ± 0.7	3.4 ± 0.5	3.4 ± 0.7
EDLVD, cm	5.0 ± 0.7	5.0 ± 0.6	4.9 ± 0.7
Primary PCI, *n* (%)	192 (95.0)	60 (92.3)	128 (97.0)

Continuous normally distributed variables are presented as mean ± standard deviation; Continuous non-normally distributed variables are presented as median [25% quartile−75% quartile]; AHI, apnoea-hypopnea-index; CAD, coronary artery disease; LAD, left anterior descending coronary artery; MI, myocardial infarction; LCX, left circumflex artery; PCI, percutaneous coronary intervention; RCA, right coronary artery;

* p < 0.05;

¶ p < 0.01;

*Mann-Whitney U test was used for non-normally distributed data*.

**Figure 2:**
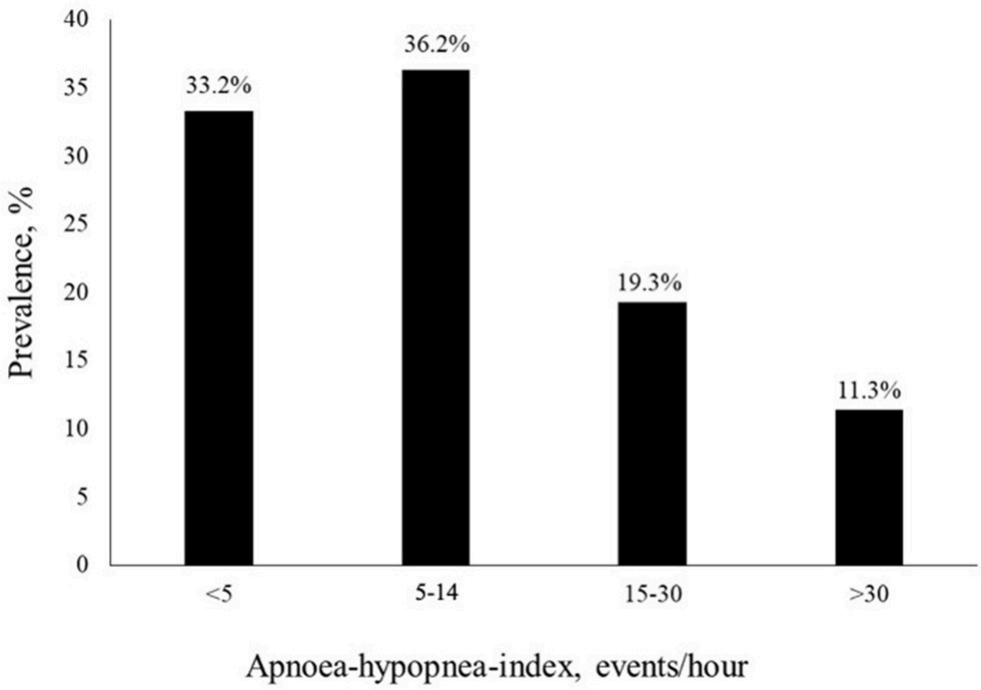
Prevalence of the different apnoea-hypopnea-index (AHI) ranges in the study collective.

Two-thirds of the study cohort (66.7%) had SDB with 44.9% of them having CSA and 21.8% OSA. Ten percentage had Cheyne-Stokes respiration pattern. The mean AHI was 13.8 1/h, the mean AI was 6.0 1/h, the mean CAI was 4.7 1/h, the OAI was 1.3 1/h, and the mean HI was 7.8 1/h. 43.4% of the study subjects had between 5 and 20 AHI events per hour. 11.4% of the SDB-group had >30 AHI events per hour. Prevalence of the different AHI-ranges is shown in Figure 2. The mean oxygen saturation was 93.0% and the lowest oxygen saturation was 81.4%. The mean ODI was 14.3 1/h. Compared to the non-SDB-group, subjects in the SDB-group had significantly lower mean oxygen saturation (92.7 vs. 93.7%), significantly higher ODI (19.3 1/h vs. 4.3 1/h) and spent significantly more time with oxygen saturation <90% (4,096 vs. 592 s). Detailed sleep study characteristics for the whole study collective, both the SDB-, and non-SDB-groups, are given in Table 3.

**Table 3:** Sleep study characteristics of the whole study collective, sleep disordered breathing (SDB)- and non-SDB-group.

	**All patients**	**Non-SDB**	**SDB**
	**(*n* = 198)**	**(*n* = 66)**	**(*n* = 132)**
AHI, events per hour	8.4 [4.0–19.5]	3.0 [2.1–4.0]	13.5 [8.4–25.1]^#^
AI, events per hour	1.8 [0.6–5.6]	0.5 [0.2–1.0]	3.8 [1.4–10.4]^#^
CAI, events per hour	1.1 [0.4–4.2]	0.4 [0.1–0.66]	2.3 [0.8–8.5]^#^
OAI, events per hour	0.4 [0.1–1.3]	0.1 [0.0–0.3]	0.8 [0.3–1.9]^#^
HI, events per hour	5.1 [2.9–10.2]	2.3 [1.2–3.2]	8.6 [5.1–14.4]^#^
ODI, events per hour	10.0 [4.0–19.0]	4.0 [3.0–5.3]	14.0 [9.0–24.0]^#^
Mean SpO2, %	93 [92–94]	94 [92–95]	93 [92–94]
Lowest SpO2, %	83 [80–86]	85 [83–88]	81 [77–85]^#^
Time spent with SpO2 < 90%, s	1,354 [476–3,132]	392 [198–882]	2,266 [1013–4003]^*^

Continuous non-normally distributed variables are presented as median [25% quartile−75% quartile]; AI, apnoea index; AHI, apnoea-hypopnea-index; CAI, central apnoea index; HI, hypopnea index; OAI, obstructive apnoea index; ODI, oxygen desaturation index; SpO2, peripher oxygen saturation;

* p < 0.05;

# p < 0.001;

*Mann-Whitney U test was used for non-normally distributed data*.

**Figure 3:**
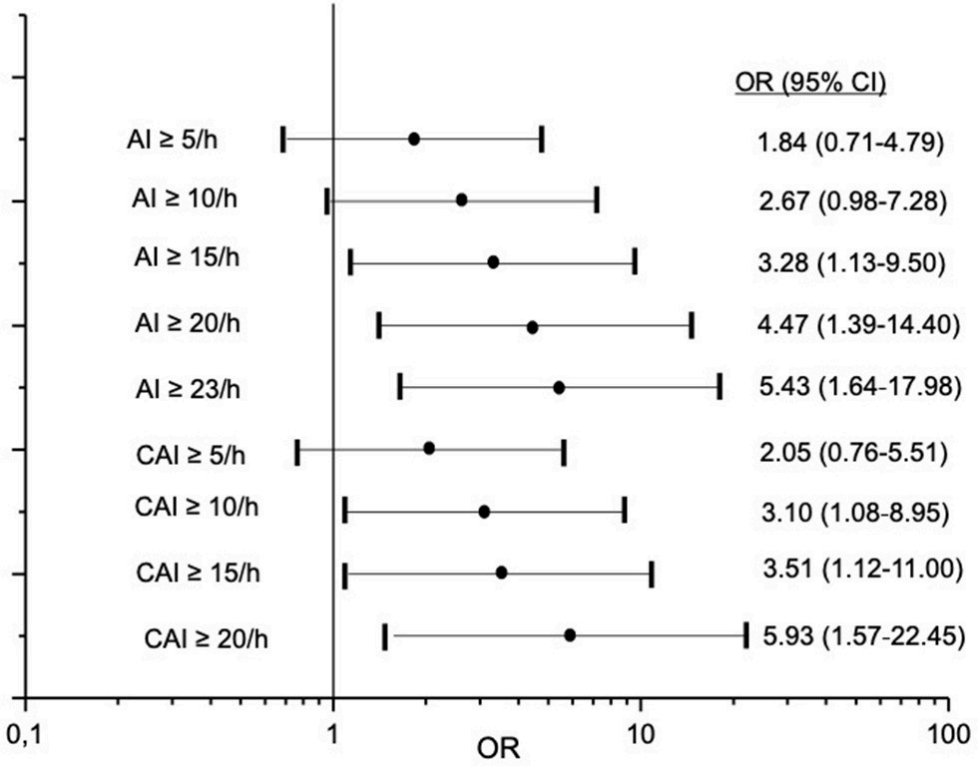
Association between different cut-off values of apnoea index (AI) and central apnoea index (cAI) and risk for non-sustained ventricular tachycardia. OR-odds ratio; CI-confidence interval.

**Figure 4:**
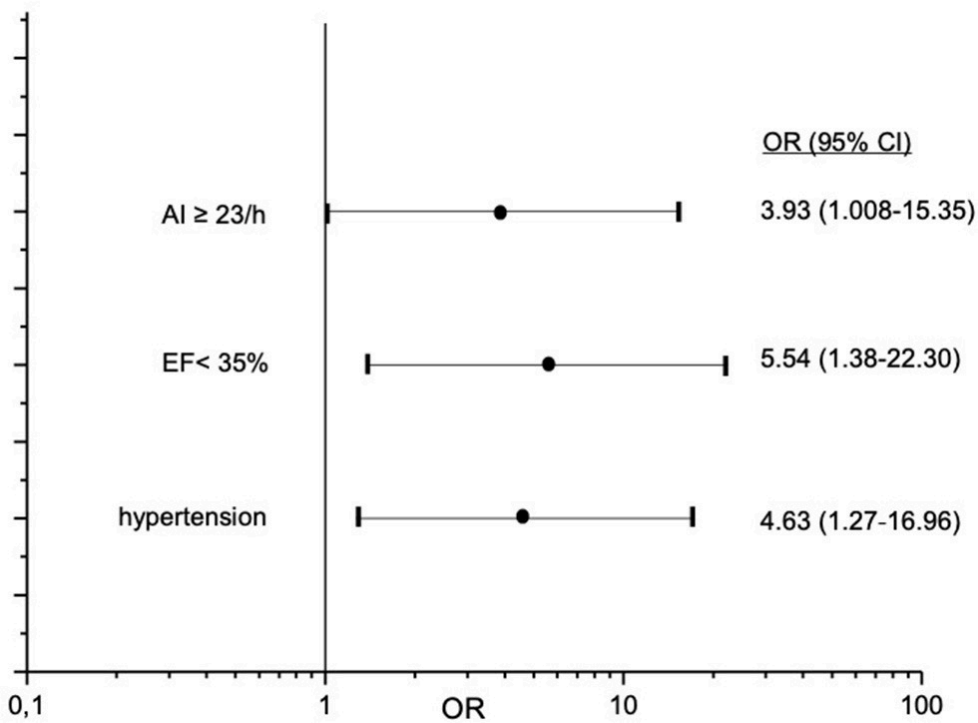
Independent predictors for occurrence of non-sustained ventricular tachycardia in study patients. OR-odds ratio; CI-confidence interval.

Valid overnight polygraphy study and Holter monitoring were available in 193 patients. The incidence of atrial fibrillation was 1%. We observed no sustained VT or ventricular fibrillation and no high-grade AV block and asystole episodes during Holter monitoring in any patient. nsVT was detected in 20 patients. Comparing to patients without nsVT, patients with nsVT had a significantly higher incidence of hypertension, diabetes mellitus, and significantly lower ejection fraction. Detailed characteristics of the patients with and without detected nsVT are given in Table 4. We did not observe a statistically significant impact of AHI, oAI, or HI of any severity on nsVT. AI of 15 and more events per hour and cAI of 10 and more events per hour was statistically significant and associated with higher occurrence of nsVT in the binary regression analysis (Figure 3). We included these parameters, the hypoxia parameters as well as “hypertension,” “diabetes mellitus” and “ejection fraction,” which differed significantly between the groups in the univariate statistical analysis with and without occurred nsVT in the multiple binary regression analysis. We found a significant independent association of AI of 23 and more events per hour, LVEF <35% and hypertension with higher risk of the nsVT occurrence (Figure 4).

**Table 4:** Baseline, cardiovascular and sleep study characteristics of the patients with and without non-sustained ventricular tachycardia (nsVT).

	**no nsVT (*n* = 173)**	**nsVT (*n* = 20)**
Male, *n* (%)	128 (74.0)	15 (75.0)
Age, years	59.7 ± 10.7	64.1 ± 9.1
BMI, kg/m^2^	27.8 ± 3.9	27.9 ± 4.2
Previous MI, *n* (%)	21 (12.2)	1 (5.0)
Hypertension, *n* (%)	95 (54.9)	17 (85.0)^*^
Diabetes mellitus, *n* (%)	25 (14.5)	8 (40.0)^¶^
Current Smoker, *n* (%)	100 (57.8)	11 (55.0)
Hyperlipidaemia, *n* (%)	119 (68.8)	14 (70.0)
1-vessel-CAD, *n* (%)	53 (30.6)	4 (21.1)
2-vessel-CAD, *n* (%)	61(35.3)	6 (31.6)
3-vessel-CAD, *n* (%)	59 (34.1)	9 (47.3)
Ejection fraction, %	57 [50–63]	51 [45–59]
Systolic diameter of the left chamber, cm	3.3 [2.9–3.7]	3.5 [3.2–4.1]
Diastolic diameter of the left chamber, cm	5.0 [4.5–5.4]	5.0 [4.3–5.4]
AHI, events per hour	8.4 [4.0–19.0]	15.1 [3.5–32.0]
AI, events per hour	1.7 [0.6–5.1]	2.8 [0.9–25.0]
cAI, events per hour	1.0 [0.3–3.6]	1.7 [0.6–20.0]
oAI, events per hour	0.4 [0.1–1.3]	1.0 [0.2–2.3]
HI, events per hour	5.5 [3.0–10.2]	4.4 [2.3–12.4]
ODI, events per hour	10.0 [4.3–19.0]	13.0 [3.8–28.3]
Mean oxygen saturation, %	93 [92–94]	94 [93–95]
Lowest desaturation, %	83 [80–86]	82 [82–85]

Continuous non-normally distributed variables are presented as median [25% quartile−75% quartile]; AHI, apnoea-hypopnea-index; AI, apnoea index; BMI, body mass index; CAD, coronary artery disease; cAI, central apnoea index; HI, hypopnea index; MI, myocardial infarction; oAI, obstructive apnoea index; ODI, oxygen desaturation index;

* p < 0,05;

¶ p < 0,01;

*Mann-Whitney U test was used for non-normally distributed data*.

LVEF was statistically significant and inversely proportionally associated with AI and cAI (Pearson correlation index −0.16 [*p* = 0.026] and −0.17 [*p* = 0.017]. There was no correlation between LVEF and AHI and oAI. Mean cAI was 4.0 ± 7.2 1/h in patients with LVEF ≥ 50% and 6.2 ± 12.0 1/h in patients with EF <50% (*p* = 0.89).

## Discussion

We found high prevalence of SDB in the subacute phase after uncomplicated AMI, which was markedly increased compared to the general population (23). Similarly, high SDB prevalence has been already observed in previous studies reporting data of patients in the subacute phase after AMI (6–9, 24, 25). Noteworthily, a comparison between different studies in terms of reported SDB prevalence is often difficult, because discrepant definitions for SDB, and its severity have been used. The predominant SDB type in our study collective was CSA. Existing scientific literature describing prevalence of CSA after AMI is limited, mainly because the cardiorespiratory sleep monitoring devices used did not differentiate between the different types of SDB (6, 15, 16). Available studies reported either a similar prevalence of CSA and OSA (6, 26) or a predominance of OSA (15). Only few studies have reported CSA as a predominant SDB type after AMI or acute coronary syndrome (27, 28). CSA is common in patients with congestive heart failure and mainly correlated with significantly impaired LVEF. Previous studies demonstrated attenuation of CSA severity when LVEF improved (29–31). Based on the findings, impaired LVEF could be a major reason for the observed high CSA prevalence in our study collective. However, several pathophysiological mechanisms in the post-AMI period beyond the impairment of LVEF could cause CSA pulmonary venous congestion, which can contribute to dilated, and disturbed answer of chemical breathing controller on changes in homeostasis (32), increased sympathetic activity (27), and impaired autonomic cardiac nerve activity (13, 32, 33). Autonomic alterations are associated with SDB, which play an important role in the developing of atrial cardiomyopathy that may represent a pathogenic factor for occurrence of ischemic stroke (34). Although we detected a statistically significant inversely proportional association between LVEF and CAI consistent with the findings of the afore mentioned studies, the mean CAI did not differ significantly between patients with preserved or mildly-impaired LVEF compared to patients with moderate to severe-impaired LVEF in our study. This is a novel finding suggesting that pathophysiological changes occurring after AMI could cause SDB and in particular CSA independent from the LVEF impairment.

Though the prevalence of SDB after AMI is high, studies investigating its impact on cardiovascular outcomes are scarce. We observed statistically significant independent impact of severe CSA on the prevalence of nsVT in the subacute phase after uncomplicated AMI with preserved mean LVEF. Preserved LVEF and revascularized culprit vessel would be possible reasons for the very low incidence of atrial fibrillation and absence of sustained VT, ventricular fibrillation, or high-grade bradycardic arrhythmias in our study collective. The presence or severity of OSA had no impact on the prevalence of nsVT. These are novel findings, which have not been reported. Our results suggest that only severe SDB is associated with higher arrhythmogenesis in the subacute phase after AMI. In our study, only 15 patients (7.4%) would be at higher risk for nsVT occurrence, when we consider the observed cut-off value of AI = 23/h as an independent risk margin. The exact mechanism linking SDB with arrhythmogenesis remains to be elucidated. However, several pathophysiologic considerations suggest possible link between CSA and arrhythmias: periodic alterations in ventilation promote oscillations in blood pressure and heart rate (35), which can promote higher cardiac workload and arrhythmias. Periodic hypoxia during and after the apnoea phases causes an increase in sympathetic nerve activity, which is a potentially arrhythmogenic feature (36). Hypoxia itself as a consequence of SDB has been shown to be associated with cardiac arrhythmias (37). Moreover, patients with CSA hyperventilate, causing hypocapnia (38), and sympathetic nerve activity increases more during hypocapnic hypoxia compared to isocapnic hypoxia (39). Acknowledging the possible important role of hypoxia in arrhytmogenesis, we analyzed the impact of hypoxia parameters (ODI, mean saturation level, and highest desaturation, time with saturation below 90%) on nsVT. We did not observe any significant association between the hypoxia parameters, and nsVT in our study collective. Arousals contribute to autonomic disturbance and enhance sympathetic activity (40). Some evidence points to the deterioration of cardiac hemodynamics caused by CSA although the association is not as clear as in OSA, which generates negative intrathoracic pressure. Yagishita-Tgawa et al. observed more paroxysmal nocturnal dyspnea and increased levels of atrial natriuretic peptide in patients with heart failure and predominant CSA compared to those with predominant OSA (41). Previous studies demonstrated the association between severe SDB and the prevalence of nsVT not only in patients with congestive heart failure or already known SDB, but also in the general population (42–44). Our results suggest the possible association of severe SDB with arrhythmogenesis beyond the impact of impaired LVEF in a collective of patients with uncomplicated AMI, which has not been described so far. Therefore, our data support the afore mentioned pathophysiologic considerations regarding the link between CSA and arrhythmogenesis and extend the evidence as the majority of available data come from patients with congestive heart failure. Thus, it would be a reasonable consideration to screen patients after AMI for SDB and–in case severe SDB would be detected–to monitor its course and its association with arrhythmias in the long-term follow up.

### Limitations

We acknowledge several limitations of the study: PG and not polysomnography (which includes EEG monitoring) has been used in the sleep studies and the total sleep time was not measured based on detection of the sleep stages by EEG, which can potentially bias the calculation of sleep apnoea severity indices. Another major limitation is that we did not classify hypopnea events as central or obstructive, which could bias the overall prevalence rates of CSA and OSA. Though consensus definitions for obstructive and central hypopnea exist, they are sophisticated and require standardization and validation. Many indicators such as paradoxical movement, snoring, and inspiratory flattering need to be included for distinction between obstructive and hypopnea event. These indicators require visual interpretation and definition of exact cut-offs, which are not yet defined, and would increase interobserver variation significantly. Moreover, some of these factors can easily be confounded, like snoring, which can be confused with several non-snoring sounds, and change in distance between sensor and nose/mouth. In this context and acknowledging the low median CAI of the study population, the observed significant impact of CAI on arrhythmogenesis could attenuate or become non-significant once hypopneas would be classified in central, and obstructive and the prevalence rate of CSA and OSA would change.

Potential arrhythmogenic factors caused by an AMI event itself could not completely be ruled out, although fully revascularisation of the culprit vessel, and the impact of LVEF as potential important factors were considered in the analysis.

## Conclusion

Prevalence of SDB is high and CSA is the predominant type of SDB in the collective of patients with uncomplicated AMI treated with modern revascularization techniques and evidence-based pharmacological therapy. Severe SDB is independently associated with a higher risk of nsVT and its course should be monitored as it is a comorbidity, which potentially has negative impact on the relevant outcomes of AMI patients. Further prospective studies are needed to assess long-term follow up of SDB after AMI, and its impact on mortality and morbidity.

## Data Availability

The datasets generated for this study are available on request to the corresponding author.

## Ethics Statement

The ethics committee of the University Hospital of Lübeck, Germany approved the study. All study participants obtained written informed consent.

## Author Contributions

AR: conception of study design, acquisition, analysis and interpretation of data, drafting the article. SP: acquisition, analysis and interpretation of data. HB: analysis and interpretation of data, drafting the article.

### Conflict of Interest Statement

The authors declare that the research was conducted in the absence of any commercial or financial relationships that could be construed as a potential conflict of interest.
